# The Co-Inheritance of Alpha-Thalassemia and Sickle Cell Anemia Is Associated with Better Hematological Indices and Lower Consultations Rate in Cameroonian Patients and Could Improve Their Survival

**DOI:** 10.1371/journal.pone.0100516

**Published:** 2014-06-30

**Authors:** Maryam Bibi Rumaney, Valentina Josiane Ngo Bitoungui, Anna Alvera Vorster, Raj Ramesar, Andre Pascal Kengne, Jeanne Ngogang, Ambroise Wonkam

**Affiliations:** 1 Division of Human Genetics, Institute of Infectious Disease and Molecular Medicine (IDM), Faculty of Health Sciences, University of Cape Town (UCT), Cape Town, Republic of South Africa; 2 Department of Microbiology, Parasitology and Haematology, Faculty of Medicine and Biomedical Sciences, University of Yaoundé 1, Yaoundé, Cameroon; 3 MRC Human Genetics Research Unit), Institute of Infectious Disease and Molecular Medicine (IDM), Faculty of Health Sciences, University of Cape Town (UCT), Cape Town, Republic of South Africa; 4 Non-Communicable Diseases Research Unit, South African Medical Research Council and University of Cape Town, Cape Town, South Africa; Institut Jacques Monod, France

## Abstract

**Background:**

Co-inheritance of α-thalassemia was reported to be associated with a delayed age of disease onset among Cameroonian Sickle Cell Anemia (SCA) patients. The present study aimed to explore the correlation between α-thalassemia, hematological indices, and clinical events in these patients.

**Methods and Findings:**

We studied 161 Cameroonian SCA patients and 103 controls (59.1% HbAA) with median ages of 17.5 and 23 years. RFLP-PCR was used to confirm SCA genotype and to describe haplotypes in the *HBB-like* genes cluster. Multiplex Gap-PCR was performed to investigate the 3.7 kb α-globin gene deletions. SNaPshot PCR, capillary electrophoresis and cycle sequencing were used for the genotyping of 10 SNPs in *BCL11A*, *HMIP1/2*, *OR51B5/6* and *HBG* loci, known to influence HbF levels. Generalised linear regression models adjusted for age, sex and SNPs genotypes was used to investigate effects of α-thalassemia on clinical and hematological indices. The median rate of vaso-occlusive painful crisis and hospitalisations was two and one per year, respectively. Stroke was reported in eight cases (7.4%). Benin haplotype was the most prevalent (66.3%; *n* = 208 chromosomes). Among patients, 37.3% (*n* = 60) had at least one 3.7 kb deletion, compared to 10.9% (*n* = 6) among HbAA controls (p<0.001). Among patients, the median RBC count increased with the number of 3.7 kb deletions [2.6, 3.0 and 3.4 million/dl, with no, one and two deletions (p = 0.01)]. The median MCV decreased with the number of 3.7 kb deletion [86, 80, and 68fl, with no, one and two deletions (p<0.0001)], as well as median WBC counts [13.2, 10.5 and 9.8×10^9^/L (p<0.0001. The co-inheritance of α-thalassemia was associated with lower consultations rate (p = 0.038).

**Conclusion:**

The co-inheritance of α-thalassemia and SCA is associated with improved hematological indices, and lower consultations rate in this group of patients. This could possibly improve their survival and explain the higher proportion of α-thalassemia among patients than controls.

## Introduction

Sickle cell disease is a life-long genetic disease that begins in childhood, affecting the structure of erythrocytes. Typically, a single DNA mutation within the beta globin gene leads to a glutamic acid to valine substitution, changing normal hemoglobin (HbA) into abnormal sickle hemoglobin (HbS). In deoxygenating or dehydrating conditions, HbS polymerizes within the erythrocytes, leading to intracellular tactoids that deform the red blood cells into the characteristic sickled shape, inducing microvascular obstruction, abnormal adhesion of leukocytes and platelets, inflammation and hypercoagulation. Individuals with SCA suffer a wide range of complications: increased susceptibility to infections, chronic hemolytic anemia; recurrent periodic acute vaso-occlusive events, and chronic damage affecting almost every organ system [1].

The allele frequency of the HbAS matches the regions of highest malaria endemicity, supporting the hypothesis that HbAS confers protection against severe malaria [2]. An estimated 305 800 neonates are affected annually with nearly two-thirds of these occurring in Africa. Sickle Cell Anemia (SCA; HbSS form) is by far the most prevalent and severe form of the disease [3]. The lack of effective early detection and treatment initiatives has resulted in a high SCA-related death rate in some African countries [4]. Cameroon is a country of about 20 million inhabitants and has a population growth of 3% per annum. The country has a high carrier frequency of SCA, ranging from 8 to 34% [5]. Although Cameroon has developed a national control programme for SCA, there is not yet provision of neonatal screening, nor is there a specialized center for lifelong medical care and surveillance for this major cause of morbidity and mortality in this country [6]. There is no universal medical insurance coverage in Cameroon, and care of SCA patients is heavily dependent on families. Poverty in Cameroon affects more than 50% of the rural population and up to 30% of the urban population [7], contributing to a high burden of SCA on parents [6]. Cameroonian SCA patients can present with exceptionally severe phenotypes, as illustrated by a high rate of stroke [8] and severely impaired neurocognitive functions [9].

Although SCA is genetically characterised by a single point mutation, there are various genetic modulators that affect the phenotype of this disease, and patients can manifest with varying degrees of clinical severity [10]. Increased levels of fetal hemoglobin (HbF), and genetic loci associated with this trait, have been shown to influence the clinical severity of SCA [11]; research has recently replicated these findings in Cameroonian SCA patients [12]. In addition, co-inheritance of α-thalassemia has been associated with a milder phenotype in SCA patients, e.g. lower stoke rate [13], but could also result in the increase of vaso-occusive painful episodes [14].

There is a scarcity of data on the co-inheritance of α-thalassemia and SCA in Africa [15]; [16], [17], and surprisingly, there have been no reports on the impact of α-thalassemia on the clinical phenotype of SCA patients in Africa. SCA patients that live on the African continent, unlike e.g. African American patients, are exposed to malaria that can potentially alter the frequency of α-thalassemia [2], therefore the pattern of co-inheritance with SCA. Recently, we reported that co-inheritance of 3.7 kb α-globin gene deletion was associated with a delayed age at diagnosis and possibly improved survival of Cameroonian patients [15]. The present study aimed to explore the correlation between 3.7 kb α-globin gene deletion and hematological indices among patients and controls, and its relation to clinical severity among patients.

## Materials and Methods

### Ethical approval

The study was performed in accordance with guidelines of the Helsinki Declaration (Brazil, 2013). Ethical approval was given by the National Ethical Committee Ministry of Public Health, Republic of Cameroon (No 033/CNE/DNM/07); and the University of Cape Town, Faculty of Health Sciences Human Research Ethics Committee (HREC REF. 132/2010). Written and signed informed consent was obtained from participants who were 18 years or older, and for the children, consent was obtained from parents/guardians with an assent from the children participants older than seven years.

### Patients and clinical events

The study was conducted at the Yaoundé Central Hospital, and Douala Laquintinie Hospital in Cameroon. Socio-demographic and clinical data were collected by means of a structured questionnaire. Parents/guardians as well as adult SCA patients were interviewed; patients' medical records were reviewed, to delineate their clinical features over the past three years. In the seminal Cooperative Study in the USA, three adverse events served as proxies for severe sickle cell disease: 1) the rate of vaso-occlusive painful crisis (VOC), 2) the occurrence of stroke and 3) the rate of acute chest syndrome [18]. In Cameroon, it was virtually impossible to assess the rates of acute chest syndrome episodes retrospectively, because of the inherent difficulties in diagnosing acute chest syndromes in the settings and poor medical records. Consequently, acute chest syndromes were not considered in our evaluation. Nevertheless, we included the number of consultations and hospitalisations, as additional proxies of clinical severity. In addition, blood transfusions and any administration of hydroxyurea were recorded. VOC events were defined as episodes that could not be attributed to causes other than SCA and required hospital visits and treatment with pain killers. Anthropometric variables [Body Mass Index (BMI), and Blood Pressures (BP)] were measured upon arrival at the hospital.

The sampling was not restricted to hospital-based patients to avoid overrepresentation of the most severe phenotypes. To fulfil this goal, two SCA patient associations in Cameroon were engaged for collaboration, and additional patients were recruited during their monthly meetings. No incentive was provided for participation in the study. Only patients who had not received a blood transfusion or hospitalisation in the past 6 weeks were included, with no patient receiving hydroxyurea treatment.

The control group were randomly selected individuals (HbAS and HbAA) who were apparently healthy blood donors, and who volunteered their participation in the study. The following information was collected from control participants: a complete hematological profile (full hematological indices and Hb electrophoresis results), and minimal socio-demographic data (age and gender).

### Hematological phenotypes

Hemoglobin electrophoresis and complete routine blood count of the SCA-affected patients were conducted upon arrival at the hospital. Two methods of HbF detection were employed in this study: 1) the Alkali Denaturation Test (ADT) initially, and 2) High Performance Liquid Chromatography (HPLC), when it became available (BIORAD D-10, USA). HbF detection was performed at the hematological laboratory of the Centre Pasteur in Yaoundé. Measurements done in patients <5 years old were excluded from the analysis because HbF levels are not yet stable at this early age. ADT was used to measure HbF levels in 28% (*n* = 39) of controls and 72% (*n* = 100) of SCD patients. These two techniques displayed differences in median values (p = 0.001) with ADT yielding a median value of 11.2% compared to 6.6% for the HPLC method.

### Genotypes

DNA was extracted from peripheral blood of both patients and controls, following instructions accompanying the commercial DNA isolation kit [Puregene blood kit (Qiagen, USA)], in the molecular diagnostic laboratory, Gyneco-Obstetric and Paediatric Hospital, Yaounde, Cameroon. Genotypic analyses were performed in the Division of Human Genetics, Faculty of Health Sciences, University of Cape Town.

#### Molecular diagnostic testing for SCA (HbSS)

PCR primers were designed to optimally amplify a 770 bp segment of the β-globin gene: PCR was carried out in a thermocycler (BIORAD, USA) and analysis for the sickle-cell mutation involved restriction enzyme analysis of the PCR product, using the restriction endonuclease Dde I (GIBCO-BRL, USA). Only patients identified to be HbSS type were included in the analysis, according to a reported method [19].

#### Haplotyping of the β-globin gene cluster

Five restriction fragment length polymorphism (RFLP) regions in the β-globin genes cluster were amplified using published primers and methods to analyse the XmnI (5'Gγ), HindIII (Gγ), HindIII (Aγ), HincII (3''Ψβ), and HinfI (5'β) restriction fragments. RFLP sites and the fragments were visualised by agarose gel electrophoresis and β-globin gene haplotypes were defined by the study of the combination of the restriction sites [20].

#### Detection of 3.7 kb α-globin gene deletions

Alpha-thalassemia is caused most frequently by deletions involving one or both α-globin genes [21]. The 3.7 kb α-globin gene deletions is the most prevalent in sub-Saharan Africans, among whom point mutations has been seldom reported in α-globin gene [22]. Alpha-globin gene deletions were screened by multiplex gap-PCR following the method reported previously [23], with a few modifications: only five primers were required for the detection of 3.7 kb and 4.2 kb deletions. The Expand Long Template PCR system was utilised with buffer 3 (Roche, Mannheim, Germany), and DMSO (1.5%) (Thermo Scientific, California, USA) was added.

#### SNPs genotyping in the HMIP, BCL11A, HBG XmnI-158 and OR51B5/6 loci

In a separate study, we reported that sequence variants at *BCL11A* and *HBS1L-MYB* loci influenced HbF levels. In addition to *BCL11A* rs4671393 SNPs that was associated with wider range of hematological indices, independently of HbF levels and two SNPs in *HBS1L-MYB* that were associated with the number of hospitalisation [12]. In this paper we also investigated the effects of α-thalassemia on clinical and hematological indices, in relation to these variants. For this purpose, ten regions containing specific SNPs were amplified: viz, for the *BCL11A* locus, SNPs rs11886868 and rs4671393; for the *HMIP1/2* loci: SNPs rs28384513, rs9376090, rs9399137, rs9389269; rs9402686 and rs9494142; for the *OR51B5/6* loci: SNP rs5006884, for *HBG* loci, SNP rs7482144. PCR was performed to determine genotypes using SNaPshot multiplex ready reaction mix (Applied Biosystems, California, USA); followed by capillary electrophoresis (Applied Biosystems California, USA) and cycle sequencing (Gene Amp PCR system 9700) were used for the genotyping of the 10 selected SNPs, as previously reported. We previously reported the details of these experiments [12].

### Statistical analysis

A Hardy-Weinberg Equilibrium (HWE) test was performed on the genotype results of 3.7 kb α-globin gene deletions and the 10 selected SNPs. Observed 3.7 kb α-globin gene deletion allele frequencies in controls were consistent with HWE (χ^2^ = 2.37; p = 0.12), equally to SCA patients (χ^2^ = 1.69; p = 0.19). Two SNPs were dropped because of significant violation of HWE (rs1188686 in *BCL11A*; HWE p-value  = 0.00030; and rs9389269 in the *HBS1L-MYB* locus; HWE P-value: 0.002876). And two others SNPs were monomorphic (rs9376090 in the *HBS1L-MYB* locus, all the patients were T/T homozygous; and rs7482144 in *HBG* loci, all the patients were G/G homozygous).

Descriptive statistics was performed for all quantitative data using SPSS (IBM, USA version 21.0). The distribution of variables of interest was assessed by the Shapiro-Wilk test and informed the use of non-parametric tests to compare groups of participants (Mann-Whitney U test, median test or the Kruskal-Wallis). Additive model per copy of the α-globin gene deletions were performed, as well as multinomial, or linear logistic regression analysis incorporating SCA genotype, α-thalassemia genotype, age, gender, or clinical events. In addition, to correct for the skewness of the HbF distribution, we log10-transformed and normalized the data to obtain the quantitative trait used in the association analysis (after correcting for age, gender, and electrophoresis technique). The effects of α-thalassemia on key clinical and hematological indices were investigated in generalised linear regression models, adjusted for age, sex and six SNPs genotypes (always assuming log-additive genetic effects) using the R statistical package version 3.0.3 [06.03.2014], The R Foundation for statistical computing, Vienna, Austria). Significance was set at the 0.05 level.

## Results

### Socio-demographic data

All 161 SCA patients and 103 controls (59.1% HbAA; *n* = 55) lived in the urban and peri-urban area of Yaoundé and Douala, the two biggest cities in Cameroon; 51% (*n* = 76) of patients and 67.8% (*n* = 59) of controls were female, with a significantly higher proportion of females among controls (p = 0.043).

Patients were relatively younger than controls (p<0.001) and the median age of SCA patients was 17.5 years (25^th^ percentile = 11 years; 75^th^ percentile = 24 years). The median age of HbAS controls was 24 years (25^th^ percentile = 17.5 years; 75^th^ percentile = 26 years) and that of HbAA controls was 26.5 years (25^th^ percentile = 23.2 years; 75^th^ percentile = 30 years).

### Anthropometric variables and clinical events in SCA patients

The patients displayed median systolic and diastolic blood pressure of 108 mmHg (25^th^ percentile = 101 mmHg; 75th percentile = 116 mmHg) and 58 mmHg (25^th^ percentile = 53; 75^th^ percentile = 62.2). The median BMI was 18.2 kg/m^2^ (25^th^ percentile = 15.7 kg/m^2^; 75^th^ percentile = 21.4 kg/m^2^).

After the review and validation of clinical data/events from parents/patients' interviews and medical records, a maximum of 121 patients had data that were suitable for the analysis (Table 1). The median rate of VOC was 2 per year (25^th^ percentile = 1 per year; 75^th^ percentile = 4 per year) and the median rate of hospitalisations was 1 per year (25^th^ percentile = 0 per year; 75^th^ percentile = 2 per year) (Table 1). High rate of VOC (>3 per year among 43% patients) and relatively high rates of overt strokes (8 cases; 7.4%) were indicative of severe phenotypes among patients. Linear regression analysis incorporating the age of patient and gender does not revealed any differences in rate of VOC and hospitalisations. Though males tended to have a higher rate of VOC (Likelihood Ratio p = 0.09).

**Table 1:** Co-inheritance of SCA-alpha thalassemia and of clinical events.

		HbSS-(αα/αα)		HbSS-(αα/α3.7)		HbSS-(α3.7/α3.7)		
		N	Median (Minimum-Maximum)	N	Median (Minimum-Maximum)	N	Median (Minimum-Maximum)	P-values
**BMI (kg/m2)**		49	17.5 (12.1–26.6)	27	17.7 (12.5–26.7)	7	18.3 (17.0–23.1)	0.91
**Systolic blood pressure (mmHg)**		49	108 (86–156)	29	108 (89–135)	6	105.5 (99–116)	0.71
**Diastolic blood pressure (mmHg)**		49	56 (41–93)	29	60 (47–60)	6	60.5 (45–70)	0.62
**No. of vaso-occlusive pain crises/year**		77	2 (0–15)	35	2 (0–40)	9	1 (1–4)	0.31
**Overt Stroke**	**YES**	6	-	1	-	1	-	0.48
	**NO**	65	-	33	-	7	-	
**No. of consultations/year**		70	2 (0–12)	31	1 (0–12)	9	1 (0–4)	0.52
**No. of hospitalisations/year**		70	1 (0–10)	33	1 (0–10)	9	1 (0–9)	0.28

### Hematological indices among SCA patients and controls

Hematological indices among patients and controls are summarised in Table 2, reporting in patients, a normocytic normochromic anemia, with higher lymphocyte and platelet counts, and also a higher HbF and HbA2 levels than those of controls.

**Table 2:** Hematological indices of SCA patients and controls (HbAA and HbAS).

	HbAA		HbAS		HbSS		
**Haematological indices**	N	Median (Minimum-Maximum)	N	Median (Minimum-Maximum)	N	Median (Minimum-Maximum)	P-values
**RBC (million cells/µl)**	64	4.4	36	4.6	149	2.7	**0.01**
		(2–9.3)		(2.1–9.3)		(1.7–5.5)	
**Hb (g/dl)**	65	13.2	38	12.7	150	7.7	**<0.0001**
		(7.9–19.5)		(6.9–18.7)		(3.3–14.5)	
**MCV (fL)**	65	81	38	79	150	82	**0.021**
		(64–95)		(56–102)		(66–112)	
**CCMH (g/dl)**	64	36.3	36	33.8	148	33.9	**0.021**
		(28.6–45.7)		(29.1–45.2)		(26.6–54.3)	
**WBC (X10^9^/L)**	65	5.1	38	4.8	150	12.4	**<0.0001**
		(4.4–6.0)		(2–19)		(2.9–42.4)	
**Lymphocyte count (X10^9^/L)**	64	2.1	38	2.2	130	5.1	**<0.0001**
		(2.8–24.4)		(0.2–9)		(1.2–21.6)	
**Monocyte count (X10^9^/L)**	64	0.6	38	0.5	130	1.2	**0.001**
		(0.4–0.8)		(0.3–2.3)		(0.4–7.8)	
**Platelet level (X 10^9^/L)**	65	224	29	219	149	354	**0.001**
		(174–651)		(137–371)		(110–802)	
**HbF (%)**	39	5.9	17	7.3	143	11.1	**0.002**
		(0–21.6)		(0–17.1)		(0–29.3)	
**HbA2 (%)**	58	3.2	32	2.8	147	3.8	**0.003**
		(0.1–5)		(0–7.2)		(1.6–18.2)	

### Haplotypes in the β-globin-like genes cluster among SCA patients

*HBB* gene haplotype data revealed the following frequencies, per number of chromosomes: Benin (66.3%; *n* = 208), Cameroon (21%; *n* = 66), atypical (11.1%; *n* = 35), Bantu (1.3%; *n* = 4), and Arab/Saudi-Indian (0.3%; *n* = 1). No Senegal haplotype was found. In combination, the Benin/Benin (42.2%; *n* = 71), Benin/Cameroon (26.8%; *n* = 42), Benin/atypical (14%; *n* = 22) and Cameroon/Cameroon (5.1%; *n* = 8) haplotypes were the most prevalent. There were no significant differences, when studying the association among the main groups of haplotype combinations and the hematological indices, clinical events or HbF levels (data not shown).

### Prevalence and allele frequency of 3.7 kb α-globin gene deletion among patients and controls

**Figure 1:**
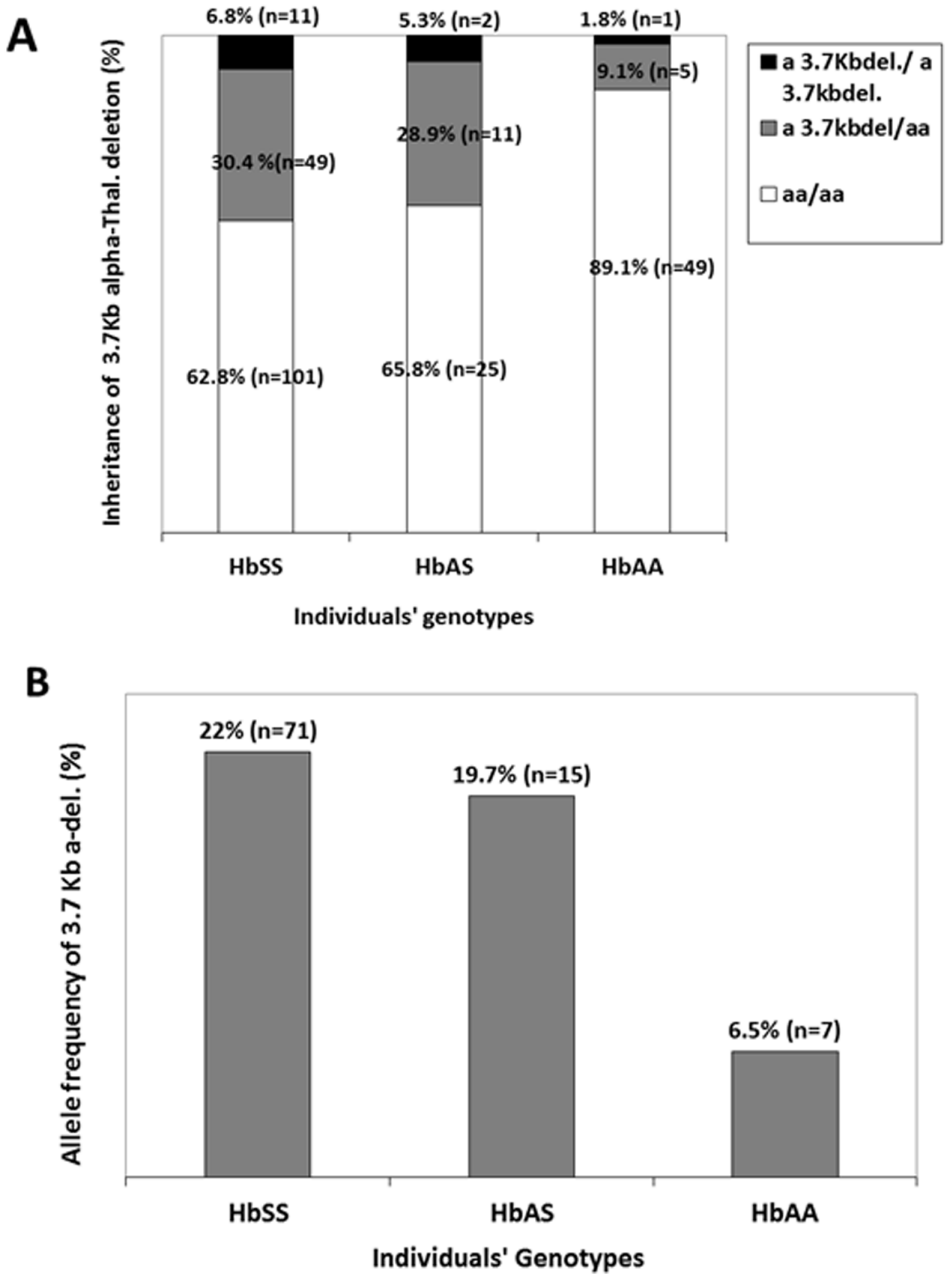
Co-inheritance of α-thalassemia among patients and controls. **Panel A** displays a much higher prevalence of 3.7α-globin gene deletions among patients compared to unaffected controls [HbAA and HbSS combined (p = 0.003)]. This difference was mostly driven by a much lower proportion of 3.7 kb α-globin gene deletions among HbAA controls. **Panel B** displays the allele frequencies of the 3.7 kb α-globin gene deletions among patient and control. The frequencies were 22% among patients and 11.8% among controls (HbAS and HbAA combined) (p = 0.006). HbAS controls had more 3.7 kb α-globin gene deletions than HbAA controls (p = 0.02).

Among controls (HbAS and HbAA), 20.4% (*n* = 19) had at least one 3.7 kb α-globin gene deletion, compared to 37.3% (*n* = 60) among patients (p = 0.007). HbAS controls had more 3.7 kb α-globin gene deletions than HbAA controls (p = 0.02) and the proportion of HbAS controls with one or two 3.7 kb α-globin gene deletions were 34.2% (*n* = 13); the proportion of HbAA controls with at least one 3.7 kb α-globin gene deletion was 10.9% (*n* = 6) (Fig 1A). Similarly, allele frequencies of the 3.7 kb α-globin gene deletions were 11.8% and 22% in controls (HbAA and HbAS) and patients (p = 0.006), respectively. Allele frequency HbAS controls of was 19.7% (*n* = 15) and that of Hb AA controls was 6.4% (*n* = 7) (Fig. 1B).

Multinomial logistic regressions analysis incorporating SCA genotype, α-thalassemia genotype, age and gender indicated that, the differential frequency of 3.7 kb α-globin gene deletions among patients and controls was mostly driven by the genotype of HbAA individuals. HbAA individuals were about 4 times less likely to have a single 3.7 kb α-globin gene deletion [exponentiation of the β coefficient (95% Confidence Interval) = 4.02 (1.45–11.13)] and 5 less likely to have a double 3.7 kb α-globin gene deletion than HbSS patients [exponentiation of the β coefficient (95% Confidence Interval) = 5.42 (0.65–44.79)].

In addition, multinomial logistic regression analysis showed that the allele frequencies among patients as compared to controls tended, non-significantly, to be influenced by gender and to a lesser extent by age. Considering sickle genotype, 3.7 kb α-globin gene genotype and gender, multinomial analysis indicated that, being male increased the likelihood of having the 3.7 kb α-globin gene deletion (Likelihood Ratio p = 0.013).

### Co-inheritance of SCA and α-thalassemia: hematological indices and clinical events

After univariate analysis, the co-inheritance of 3.7 kb α-globin gene deletion and SCA was significantly associated with a lower MCV and higher RBC, WBC, monocyte and lymphocyte counts (Table 3) and no significant differences were observed across α-thalassemia genotypes, when comparing anthropometric variables and clinical events (Table 1).

**Table 3:** Co-inheritance of SCA and α-thalassemia and hematological indices.

	HbSS-(αα/αα)		HbSS-(αα/α3.7)		HbSS-(α3.7/α3.7)		
**Haematological indices**	N	Median	N	Median	N	Median	P-values
		(Minimum-Maximum)		(Minimum-Maximum)		(Minimum-Maximum)	
**RBC (million cells/µl),**	94	2.6	46	3	10	3.4	**0.01**
		(1.7–4.7)		(1.8–5.5)		(1.9–5.5)	
**Hb (g/dl)**	93	7.7	45	8	10	8.1	0.55
		(3.4–13.2)		(4.9–14.5)		(5.4–14.3)	
**MCV (fL)**	93	86	45	80	10	68	**<0.0001**
		(66–112)		(66–100)		(59–101)	
**CCMH (g/dl)**	99	34.5	44	32.9	10	31.7	**0.01**
		(28–54.3)		(28.6–54.3)		(28.8–44.1)	
**WBC (X10^9^/L)**	100	13.4	45	10.5	10	9.7	**0.001**
		(4–42.4)		(2.9–24)		(4.1–17.4)	
**Lymphocytes count (X10^9^/L)**	80	6	39	4.2	9	2.9	**0.007**
		(1.9–21.6)		(1.8–8.4)		(1.6–8.2)	
**Monocytes count (X10^9^/L)**	80	1.5	39	1.1	10	0.8	**0.002**
		(0.4–7.8)		(0.4–3)		(0.4–2.3)	
**Platelets count (X10^9^/L)**	92	354.5	45	373	10	252	0.73
		(110–650)		(148–802)		(177–559)	
**HbF (%)**	89	12.1	42	8.8	10	14.8	0.11
		(0–29.3)		(0–26.2)		(0.8–27.3)	
**HbA2 (%)**	91	3.7	44	3.9	10	4.7	0.07
		(1.6–18.2)		(1.9–13.2)		(2.5–5.3)	

Effects of α-thalassemia on key clinical and hematological indices in generalised linear regression models, adjusted for age, sex and five SNPs that influence HbF levels are summarised on Table 4. The co-inheritance of alpha-thalassemia was associated with lower consultation rate (p = 0.038). The effects of the co-inheritance of α-thalassemia on RBC count, MCV and lymphocytes count were still observed (Table 4). Two SNPs were associated with specific hematological indices: *BCL11A* rs4671393 was significantly associated with HbF level (p = 0.005; Table 4); *HMIP* rs9399137 was significantly associated with lower lymphocyte count (estimate = −2.09816; standard deviation = 1.02912; p = 0.044) and borderline associated with lower platelets count (estimate = −76.72; standard deviation =  43.16; p = 0.078). In addition, being female was associates with higher MCV (estimate = 3.88, standard deviation = 1.73; p = 0.02), higher HbA2 (estimate = 1.02; standard deviation = 0.48; p = 0.035) and higher HbF level (estimate = 3.82; standard deviation = 1.44; p = 0.009).

**Table 4:** Effects of α-thalassemia on key clinical and hematological indices, in generalised linear regression models, adjusted for age, sex and five SNPs* that influence HbF level.

Outcomes	Unit of measurement	Single deletion vs. No deletion		Double deletion vs. No deletion		Number of Observations
		Estimates (Standard error)	p-values	Estimates (Standard error)	p-values	
**Consultations**	Day/year	−1.32 (0.63)	**0.038**	−1.31 (1.06)	0.221	110
**Hospitalisation**	Day/year	−0.17 (0.34)	0.608	−0.37 (0.57)	0.514	104
**Vaso-occlusive crisis**	Number/year	−0.57 (0.50)	0.253	−1.49 (0.84)	0.079	121
**RBC count**	million cells/µl	0.37 (0.16)	**0.021**	1.03 (0.27)	**0.0002**	149
**Hemoglobin**	g/dl	0.58 (0.37)	0.120	0.72 (0.63)	0.25	150
**HbA2**	(%)	0.23 (0.52)	0.656	0.04 (0.87)	0.956	143
**HbF**	(%)	−1.55 (1.57)	0.327	0.97 (2.64)	0.715	147
**MCV**	fl	−5.72 (1.89)	**0.003**	−18.18 (3.19)	**<0.0001**	150
**WBC count**	X10^9^/L	−3.43 (1.04)	**0.001**	−4.31 (1.75)	**0.015**	150
**Lymphocytes count**	X10^9^/L	−2.15 (0.655)	**0.001**	−2.41 (1.102)	**0.030**	130
**Monocytes count**	X10^9^/L	−0.51 (0.20)	**0.01**	−0.67 (0.34)	**0.05**	130
**Platelets count**	X 10^9^/L	−4.80 (27.49)	0.86	−45.42 (46.23)	0.32	149

*HbF related SNPs are: *BCL11A* rs4671393, *HBS1L-MYB* rs28384513, *HBS1L-MYB* rs9399137, *HBS1L-MYB* rs9402686, *HBS1L-MYB* rs9494142 and *OR51B5/6* rs5006884.

## Discussion

A high prevalence of the 3.7 kb α-globin gene deletion has also been reported among SCA patients in Brazil (29%) [24], in India (32%) [25], in the UK among African Britons (34%) [26], in Guadeloupe (36%) [27], in Saudi Arabia (40%) [28], in the USA among African Americans (41%) [29], in Oman (43%) [30], in France among Africans (48%) [31], and in Tanzania (58%) [16]. However, none of these studies compared the prevalence of 3.7 kb α-globin gene deletion to unaffected controls from the same setting. By doing this, the present study has provided a unique contribution toward consolidating the hypothesis of a possible positive effect of the 3.7 kb α-globin gene deletion on survival of SCA patients [15]. A decade ago in Congo, researchers reported a less stringent difference with 67.2% SCA patients who had co-inherited the 3.7 kb α-globin gene deletion, as compared to 54.8% of HbAA adults [17]. In Yemen, a similar trend was reported with 34.6% of SCA patients carrying the 3.7 kb α-globin gene deletion, compared to 26.3% in the HbAA group [32]. To support our findings, the prevalence of controls individual (HbAS and HbAA) who have at least one 3.7 kb α-globin gene deletion in the present study (20.4%) is comparable to that reported previously in many other settings across Africa. The prevalence of 3.7 kb α-globin gene deletion was: 15.8% in Kenya [33], 15.1% in Rwanda [34], 20.8% in Guinea (West Africa) [35], and 10%–25% in high-altitude villages in Northern Tanzania [36]. Nevertheless, comparison across populations from different ethnicities and geographical location needs some caution, as the advantageous effect of α-thalassemia is clearly associated with altitude, age of individuals and endemicity of malaria [36]. Individually, HbS and α-thalassemia, are protective against severe *Plasmodium falciparum* malaria, but, there is a possible negative epistasis between α-thalassaemia and sickle cell trait which can modulate the inter population variation [2]. Thus, the significance of a much higher prevalence of 3.7 kb α-globin gene deletion among HbAS than HbAA controls reported will require further investigations in relation to malaria protection.

Noticeably, in a seminal work in the USA, authors reported three decade ago that, in the first ten years of life among HbSS individuals, the prevalence of 3.7 kb α-globin gene deletions was comparable to that in the general African American population (17%), while after 20 years of age, the prevalence increased to 49% [37]. Equally, the prevalence of the 3.7 kb α-globin gene deletion increased with age in Cuban SCA patients [38]. In addition to the higher proportion of 3.7 kb α-globin gene deletions among patients, we reported that the co-inheritance of 3.7 kb α-globin gene deletions delayed the onset of clinical manifestations [15]. These data, in addition to previous report, are implying that co-inheritance of α-thalassemia could be associated with longer survival of SCA patients. Nevertheless, the delayed age of first symptoms may not necessarily be related to lifetime milder clinical expression. Interestingly, after multivariate analysis in the present study, the co-inheritance of α-thalassemia was shown to be associated with lower consultation rate. But, in the present first attempt in both Cameroon and Africa, we did not find any significant influence of the co-inheritance of the 3.7 kb α-globin gene deletion and other clinical events in SCA (Table 1). This is not necessarily unexpected, due to the small sample size and the challenge to define a SCA severity scoring that could be universally used [39]. Specifically, the potential deleterious effect of α-thalassemia on the number of painful episodes reported previously [14], [27], could be difficult to validate in a context, such as Cameroon, where free medical services for patient is unavailable. Pain tolerance and socio-economic factors could have influenced the number of hospital visits and biased our evaluation of clinical events. If designed appropriately, future studies in Africa could explore the potential beneficial effect of the co-inheritance of α-thalassemia and SCA, on specific phenotype such as lower hemolysis [40], lower frequency of gallstones [41], lower albuminuria [26], [29], [42] or lower risk of the occurrence of a stroke [13], [24], [43]. Noticeably, the present study confirms that the presence of 3.7 kb α-globin gene deletion improved hematological indices and mitigates the degree of anemia in SCA patients (Table 2).

Indeed, α-thalassemia has been shown to diminish the severity of disease by reducing the amount of sickled RBC, increasing the HbF level and HbA2, and decreasing the intracellular HbS level, which results in a reduction in HbS prompted cellular destruction, thereby improving hemolysis [44], [45]. The decrease in WBC was attributed to a drop in the hemolytic rate, the amount of sickled red blood cells and a reduction in the inflammatory process. The extent of the protective effect was also shown to be in accordance with the amount of α-globin genes deleted [24]. The improved hematological indices could be the major factor that contributes to ameliorate the general well-being and possibly survival of SCA patients, and ultimately explain a much higher prevalence of 3.7 kb α-globin gene deletion among patients, than HbAA controls. Nevertheless, in the present study, the results of the generalised linear regression models seem to indicate that others genomic factors as well as demographic factors could also affect the hematological indices (Table 4).

### Limitations

The first limitation of the study is the relatively small sample sizes of patients and controls that were not age gender or ethnically matched. Another methodological limitation is the limited use of the gold standard, i.e. HPLC method to measure HbF, because the ADT method is less precise [46], and this could have affected the association of haplotypes, to HbF levels and clinical events. Nevertheless, in a different study, we disaggregated SCA patients sample, based on the HbF assessment technique (ADT vs HPLC), and found that the significant associations with HbF levels, examined independently, were present in both sub-groups studied using the different assay methods, in rs4671393 (*BCL11A*), rs28384513 (*HMIP 1*) and rs9494142 (*HMIP 2*) [12]. The self-reported nature of clinical variables such as VOC episodes limits the interpretation of the results, as pain tolerance and the financial status could have been modifying factors for hospital attendances.

### Practical implications and perspectives

The possibility that α-thalassemia could have a strong effect on survival of SCA patients, offers the prospect of profiling patients from birth and addressing a closer follow up. To define a global severity scale for the purpose of genomic studies of SCA is challenging; however, some preliminary data are encouraging. Indeed, in a recent study in Cameroon, two SNPs in *HBS1L-MYB* that influence HbF level were also associated with the number of hospitalisations and in the present study the co-inheritance of α-thalassemia was associated with lower consultation rate. If confirmed, these data could add to the evidence of the clinical effects that are associated with these variants, and a clue on how to measure them. It will be interesting in future on a much larger sample size, to study the concurrent effects of the various genomic loci that influence HbF level, α-thalassemia, socio-demographic and environmental factors such as bacteraemia and malaria, on the survival of patients, in both urban and rural settings.

Despite the fact that more than 70% of SCA sufferers live in Africa, most advances in the molecular understanding and management of SCA have been based on research conducted in the USA or Europe. In the context of Africa, SCA could be consider a neglected tropical disease [47]; thus, the capacity-building dimension that this study provides is worth to be underlined, as it was completely performed on the African continent and could create further research opportunities.

## Conclusion

The study confirmed the co-inheritance of α-thalassemia with improved hematological indices and lower consultations rate, which could contribute ameliorate the general well-being and possibly the survival of patients; and ultimately explain the higher proportion of 3.7 kb α-globin gene deletion among SCA patients than controls, specifically HbAA individuals.
